# Non-adiabatic effects in the photodissociation of H_2_S

**DOI:** 10.1039/d6cp02139e

**Published:** 2026-08-25

**Authors:** Armando N. Perri, Alexander O. Mitrushchenkov, Sergei N. Yurchenko, Jonathan Tennyson

**Affiliations:** a Department of Physics & Astronomy, University College London London WC1E 6BT UK j.tennyson@ucl.ac.uk; b MSME, Université Gustave Eiffel, CNRS UMR 8208, Univ Paris Est Creteil F-77474 Marne-la-Vallée France

## Abstract

The photodissociation of triatomic molecules is relevant to the atmospheric chemistry of terrestrial and astronomical systems, where H_2_S photodissociation influences the non-equilibrium chemical networks of sulphur-rich environments. This work presents photodissociation cross sections for H_2_S in its first electronic absorption band at temperatures 1 and 295 K as computed within a time-independent variational framework. The calculations are performed in the adiabatic and diabatic representations with modified *ab initio* surfaces available in the literature using an updated version of the EVEREST nuclear motion program. It is demonstrated that the inclusion of non-adiabatic effects, associated with conical intersections between the excited 1 ^1^A_2_ (1 ^1^A″) and 1 ^1^B_1_ (2 ^1^A″) electronic states, is essential to correctly model the low-energy wing of the spectrum. The new diabatic treatment thus leads to significantly improved agreement with measured data at 295 K, especially in the important low-energy threshold region, in comparison with the uncoupled adiabatic model. The methodology established herein provides a foundation for future temperature-dependent photodissociation calculations of H_2_S and other triatomic molecules with coupled electronic states.

## Abstract 2

Photodissociation cross sections calculated for the first electronic absorption band of H_2_S at 295 K using the adiabatic and diabatic representations compared with the experimental measurements.

## Graphical abstract

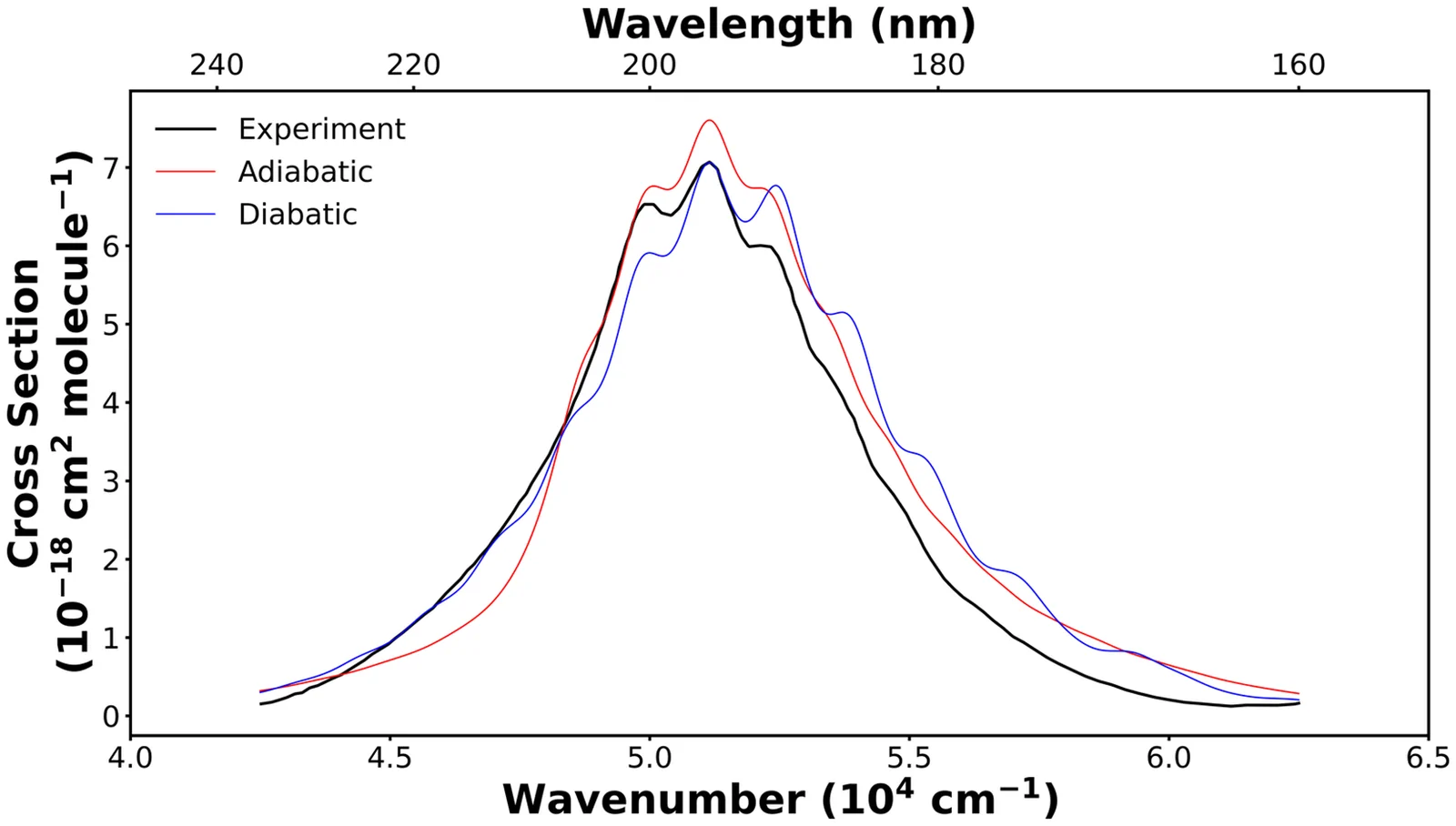

## 1 Introduction

Photodissociation is the chemical phenomenon of molecular fragmentation initiated by the absorption of a photon.^1^ Such photolysis processes have long been known to be essential in both terrestrial^2,3^ and astronomical^4–6^ environments, which drive conditions away from equilibrium to increase chemical diversity and modify overall thermal structure.

The photodissociation of triatomic molecules is of particular theoretical interest. In contrast to diatomic molecules, triatomics possess several dissociation channels for a given electronic state, and can exhibit pronounced resonance structure associated with excitation in non-dissociative degrees of freedom. An accurate description of dissociation using multi-dimensional surfaces and an exact kinetic energy operator thus poses a significant challenge, especially when strong couplings (*e.g.* spin–orbit, non-adiabatic, Renner–Teller) exist between electronic states.

The prototypical mechanism of photodissociation occurs in the first electronic absorption band of water (H_2_O),^7–11^ which proceeds as

1 H_2_O (X̃ ^1^A_1_) → H_2_O (Ã ^1^B_1_) → OH + H

**Fig. 1:**
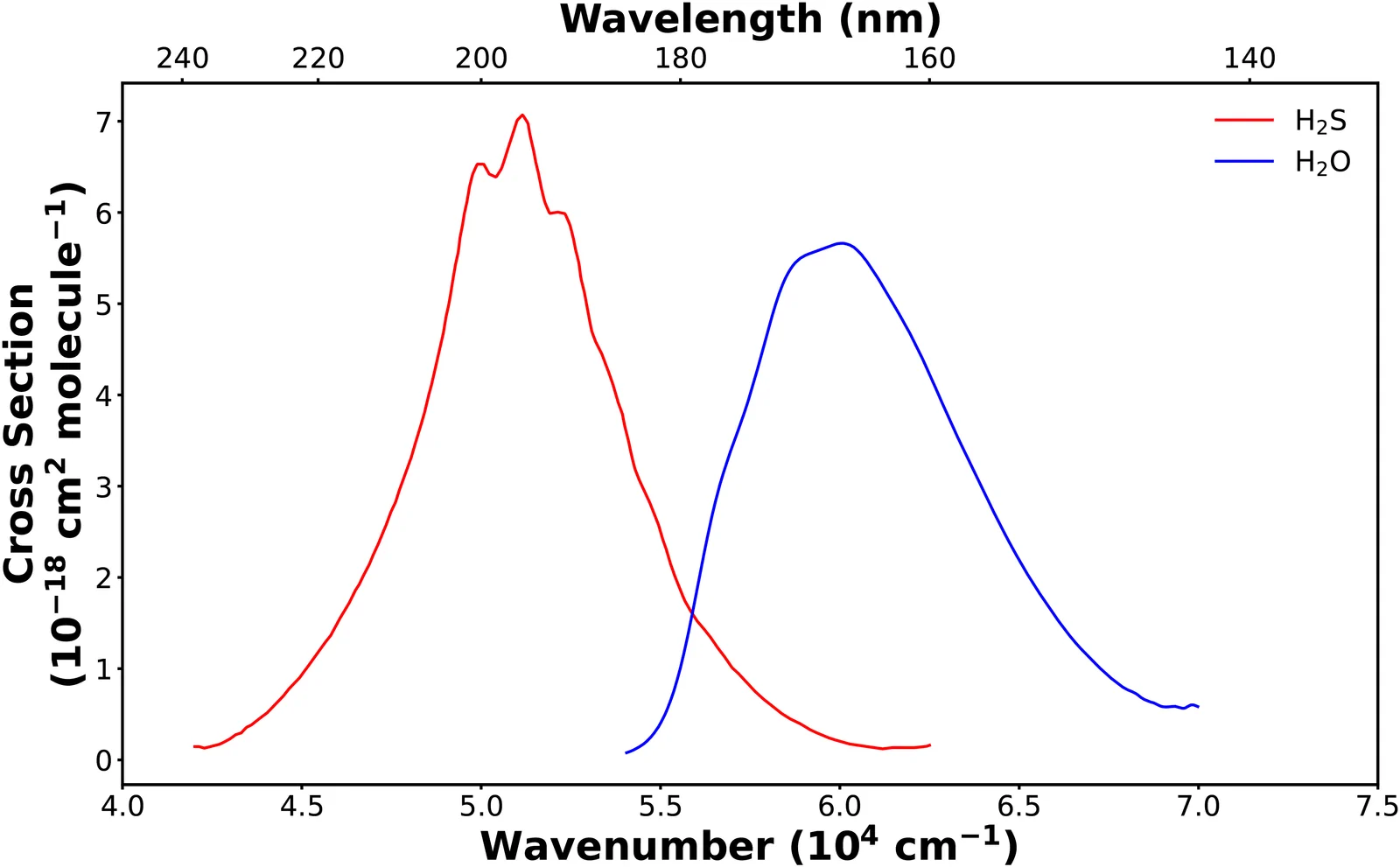
The experimental photodissociation cross sections for H_2_O and H_2_S in their first electronic absorption band as included in the Leiden database.^5,6^ These spectra were measured by Mota *et al.*^12^ and Lee *et al.*,^13^ respectively.

The process (1) for H_2_O involves excitation from the ground X̃ ^1^A_1_ (1 ^1^A′) electronic state to the excited repulsive Ã ^1^B_1_ (1 ^1^A″) allowing for its dissociation, where the labels indicate symmetry in *C*_2v_ (*C*_s_). This type of photodissociation is fast and direct on the single excited surface, which is characteristic of the broad cross section shown in Fig. 1. There are very diffuse ridge-like features due to symmetric stretch motion in the upper surface.

A comparison can then be made to the heavier homologue of H_2_O, hydrogen sulphide (H_2_S). Although the cross sections for both molecules in Fig. 1 are similar, the first electronic absorption band of H_2_S instead proceeds as

2 H_2_S (X̃ ^1^A_1_) → H_2_S (1 ^1^B_1_) → H_2_S (1 ^1^A_2_) → SH + H

The process (2) for H_2_S involves excitation from the ground X̃ ^1^A_1_ (1 ^1^A′) electronic state first to the excited bound 1 ^1^B_1_ (2 ^1^A″) electronic state, which then undergoes predissociation *via* the excited repulsive 1 ^1^A_2_ (1 ^1^A″) electronic state. This two-state mechanism was first proposed by Heumann *et al.*^14^ and supported in later studies.^15,16^ This superseded a prior proposal of Dixon *et al.*^17^ claiming that the fragmentation proceeds directly *via* the 1 ^1^A_2_ state as for H_2_O, with the diffuse structures arising from couplings to the 1 ^1^B_1_ state. In *C*_2v_ symmetry, the optically-bright 1 ^1^B_1_–X̃ ^1^A_1_ band is allowed by electronic dipole selection rules, whereas the optically-dark 1 ^1^A_2_–X̃ ^1^A_1_ band is strictly forbidden. Nonetheless, there are strong non-adiabatic interactions between the 1 ^1^A_2_ and 1 ^1^B_1_ states away from *C*_2v_ geometries, where their potential energy surfaces exhibit a seam of conical intersections throughout the coordinate space. There is notably an allowed *C*_2v_ crossing in the Franck–Condon region, which results in significant coupling at asymmetric geometries in its vicinity.

The first electronic absorption band of H_2_S is thus a useful system to assess both electronic structure and nuclear motion theory. In the literature, excited electronic states of H_2_S have been routinely investigated with subsequent spectral simulation.^14–25^ These calculations are widely compared to the experimental cross section of Lee *et al.*^13^ measured at 295 K as shown in Fig. 1. This cross section is included in the Leiden Observatory photodissociation and photoionisation database,^5,6^ and also used here for comparison. Again, unlike H_2_O, the first electronic absorption band of H_2_S shows pronounced resonance structure, allowing for vibrations along the symmetric stretch coordinate to be probed in addition to the photodissociation dynamics.

The well-established approach to computing photodissociation cross sections is to solve the time-dependent nuclear Schrödinger equation.^26^ Such calculations were performed, for example, by Heumann *et al*.,^14^ Simah *et al.*,^15^ and Chen *et al.*^16^ to model the first electronic absorption band of H_2_S. These time-dependent studies typically only propagate wavepackets corresponding to the lowest few energy levels, which limits extension to elevated temperatures. For H_2_S, excitation is only typically considered from the lowest *para* (*J*_*K*_a_*K*_c__ = 0_00_) and *ortho* (1_01_) states of the ground X̃ ^1^A_1_ (1 ^1^A′) electronic state, which have an energy separation of 13.7 cm^−1^ and spin statistical population ratio of 1 : 3.

On the other hand, the time-independent nuclear Schrödinger equation may be solved. This has the main advantages of being complete, state-selective, and capable of treating resonances (see *e.g.* Pezzella *et al.*^27^). In the approach of Pezzella *et al.*,^28^ the time-independent nuclear Schrödinger equation is solved within a finite radial box. This yields discrete energy levels that sample the continuum, where *ad hoc* line broadening must be applied to produce a continuous cross section. For polyatomic molecules, this time-independent variational framework has already been explored for HCN,^27^ however, has not yet been used to treat any species with multiple electronic states coupled by non-adiabatic effects. As such, the principal objective of this work is to extend the triatomic nuclear motion program EVEREST^29^ to treat a coupled model within a diabatic representation.

The ExoMol project^30–33^ aims to generate variational (calculated) molecular line lists and cross sections, as well as further spectral and thermodynamic data, for the study of exoplanet and other hot atmospheres. In recent years, careful attention has been given to the calculation of temperature-dependent continuum photoabsorption and photodissociation cross sections. ExoMol relies on solution of the aforementioned time-independent nuclear Schrödinger equation using variational programs, notably Duo,^34^ and EVEREST.^29^ The ExoPhoto database,^35^ a subsidiary of ExoMol, currently provides temperature-dependent photodissociation cross sections for HF, HCl,^36^ OH,^37,38^ NH,^39,40^ CH,^41,42^ and SO^43^ calculated by the ExoMol group. A temperature-dependent treatment of the uncoupled triatomic molecule HCN,^27^ as well as the diatomics CN and H_2_, are also in progress. Accordingly, this work extends the time-independent variational approach originally proposed by Pezzella *et al.*^28^ for triatomic molecules to treat diabatic potential energy surfaces characterised by strong non-adiabatic effects. The resulting photodissociation cross sections are investigated here at temperatures 1 and 295 K to test this new theoretical implementation.

This article is structured as follows: the methodologies associated with the electronic structure, nuclear motion and spectral calculations are outlined in Section 2. The photodissociation cross sections calculated in the adiabatic and diabatic representations, along with a discussion on convergence and the quality of *ab initio* surfaces, are presented in Section 3. An outlook on future temperature-dependent calculations for H_2_S, as well as for other triatomic molecules with coupled electronic states, is given in Section 4.

## 2 Methodology

### 2.1 Electronic structure calculations

The surfaces used in this work were adapted from Azzam *et al.*^44^ and Chen *et al.*,^16^ which both performed electronic structure calculations in Molpro.^45,46^ For the ground X̃ ^1^A_1_ electronic state, Azzam *et al.*^44^ calculated *ab initio* data at the CCSD(T) level of theory with the aug-cc-pV(Q+d)Z basis set. For treatment of the excited 1 ^1^A_2_ and 1 ^1^B_1_ states, Chen *et al.*^16^ employed the SA-CASSCF/icMRCI-F12 level of theory^47–50^ with the cc-pVQZ-F12 basis set.^51^

#### 2.1.1 Potential energy surfaces

The empirical PES-Y0125 was presented by Azzam *et al.*^44^ for the ground X̃ ^1^A_1_ electronic state. This was constructed by refining the underlying *ab initio* surface to experimental energy levels for *J* = 0, 1, 2 and 5. This reproduces rovibrational levels for *J* ≤ 10 with a standard deviation of 0.11 cm^−1^. The analytic functional form used is given by3
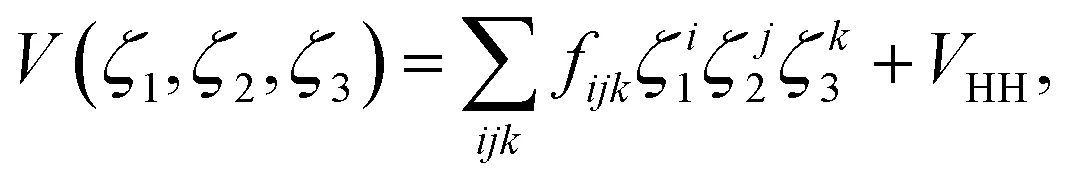
with maximum expansion order *i* + *j* + *k* = 8. The vibrational coordinates are

4 *ζ*_1_ = 1 −*e*^−*β*(*r*_1_−*r*_1*e*_)^,

5 *ζ*_2_ = 1 −*e*^−*β*(*r*_2_−*r*_2*e*_)^,

and

6 *ζ*_3_ = cos(π − *θ*_*e*_) − cos(π − *θ*),

where *r*_1_ = *r*_SH_1__, *r*_2_ = *r*_SH_2__ and *θ* = ∠H_1_SH_2_. The two radial coordinates *ζ*_1_ and *ζ*_2_ represent extended Morse oscillators, and *V*_HH_ is a hydrogen–hydrogen repulsion term given by

7 *V*_HH_ = *B*_1_*e*^−*g*_1_*r*_HH_^ + *B*_2_*e*^−*g*_2_*r*^2^_HH_^.

PES-Y0125 was used to calculate rovibrational spectra for temperatures up to 2000 K. These have been successfully utilised in a range of astronomical studies, particularly in the analysis of exoplanet atmospheres with JWST.^52–54^ This potential energy surface will thus be used herein over an *ab initio* surface due to its high-accuracy reproduction of experimental energy levels.

**Fig. 2:**
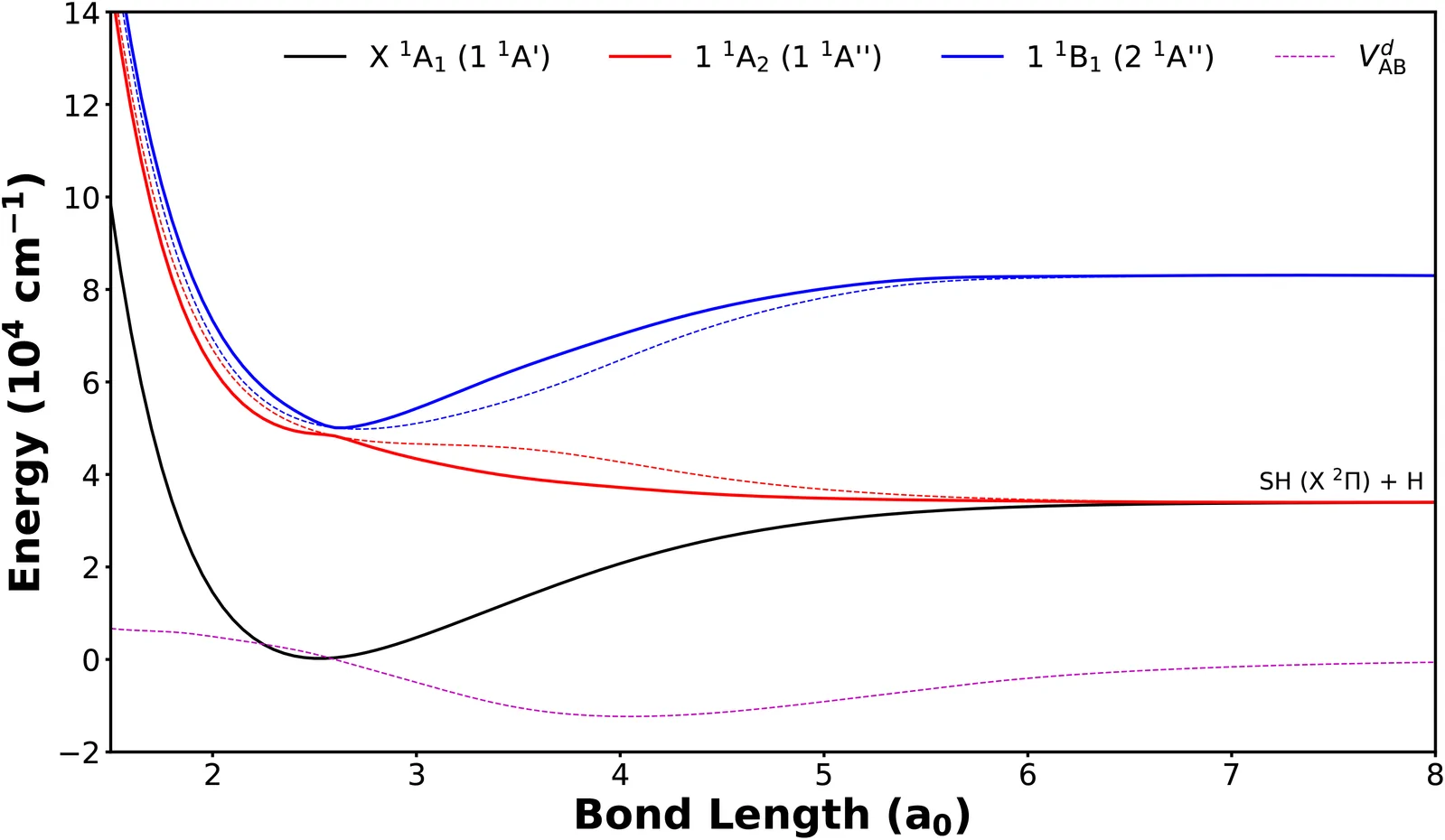
A slice of the X̃ ^1^A_1_ (1 ^1^A′), 1 ^1^A_2_ (1 ^1^A″) and 1 ^1^B_1_ (2 ^1^A″) potential energy surfaces along the *r*_2_ coordinate with *r*_1_ = 2.6 *a*_0_ and *θ* = 92° from Chen *et al*.^16^ The solid and dashed lines give the surfaces in the adiabatic and diabatic representations, respectively. The dashed magenta line gives the off-diagonal diabatic potential energy surface.

For the excited 1 ^1^A_2_ and 1 ^1^B_1_ electronic states, Chen *et al.*^16^ recently generated fitted potential energy surfaces using a neural network of permutationally invariant polynomials (PIPs) formed from symmetrised monomials of Morse-like variables. These were fitted in the diabatic representation by transforming the *ab initio* data using the procedure of Simah *et al.*^15^ A slice of these surfaces along the *r*_2_ bond length, with fixed *r*_1_ = 2.6 *a*_0_ and *θ* = 92°, is shown in Fig. 2. It was found, however, that these surfaces possess holes with negative energy for small bond angles. This is problematic in variational calculations, so this region was patched using the hydrogen–hydrogen repulsion term of Azzam *et al.*^44^ given in eqn (7). For nuclear configurations with ground state energy below 5000 cm^−1^, this repulsion term does not exceed 2 cm^−1^ at the inner wall of each PES, and becomes negligible in dissociative regions when one hydrogen atom is extended.

In contrast to the ground X̃ ^1^A_1_ electronic state, the potential energy surfaces for the excited 1 ^1^A_2_ and 1 ^1^B_1_ states exhibit complicated topologies due to conical intersections. This leads to strong non-adiabatic effects, which may be minimised by transforming from the adiabatic to (quasi-)diabatic representation. For a two-state system, the adiabatic and diabatic representations are related by8
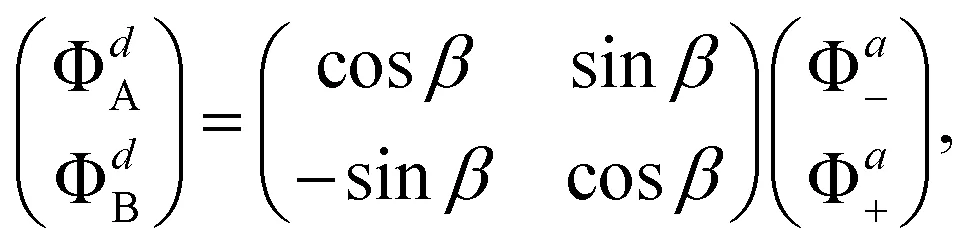
where *Φ*^*d*^_A_ and *Φ*^*d*^_B_ are the diabatic electronic wavefunctions, *Φ*^*a*^_±_ are the adiabatic electronic wavefunctions and *β* is the mixing angle. These each exhibit a parametric dependence on the nuclear coordinates. For the potential energy surfaces, the adiabatic-to-diabatic transformation yields

9 *V*^*d*^_A_ = *V*^*a*^_−_cos^2^*β* + *V*^*a*^_+_sin^2^*β*,

10 *V*^*d*^_B_ = *V*^*a*^_−_sin^2^*β* + *V*^*a*^_+_cos^2^*β*,

and11
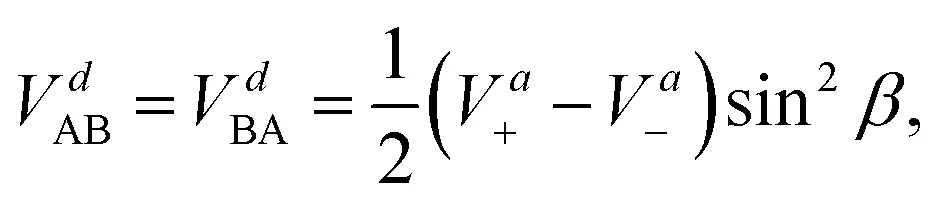
where *V*^*d*^_A_ and *V*^*d*^_B_ are the diabatic potential energy surfaces, *V*^*a*^_±_ are the adiabatic potential energy surfaces and *V*^*d*^_AB_ is the diabatic coupling surface. As non-adiabatic couplings are transformed to an off-diagonal (scalar) coupling surface in the diabatic representation, which is absent in the adiabatic representation, it is also true that *V*^*a*^_±_ are the eigenvalues of the matrix12
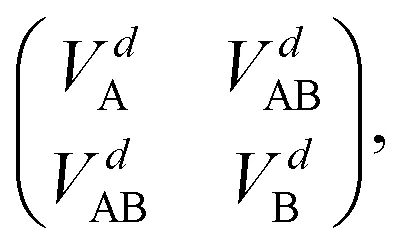
which yields13
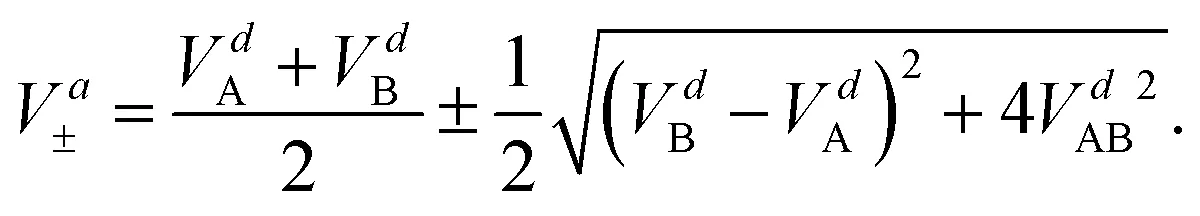


For diatomic molecules, the two-state symmetric adiabatic-to-diabatic transformation above is exact as integration is performed along one dimension and therefore path-independent.^55^ For polyatomic molecules, however, the transformation is neither unique nor exact for all nuclear geometries.^56^ Nevertheless, the (quasi-)diabatic representation allows for a robust inclusion of non-adiabatic effects without introduction of coupling terms in the kinetic energy operator, which become singular in the limit of an avoided crossing.^57^ The mixing angle determined by Chen *et al.*^16^ follows the implementation of Simah *et al.*^15^ in Molpro, where unitary transformation of the active orbitals minimises their geometry dependence with respect to a reference *C*_2v_ geometry.

#### 2.1.2 Dipole moment surfaces

The 1 ^1^A_2_–X̃ ^1^A_1_ and 1 ^1^B_1_–X̃ ^1^A_1_ transition (off-diagonal) dipole moment surfaces are required to model the first electronic absorption band of H_2_S. These transform between the adiabatic and diabatic representations as

14 *μ*^*d*^_A_ = *μ*^*a*^_−_cos *β* + *μ*^*a*^_+_sin *β*

and

15 *μ*^*d*^_B_ = −*μ*^*a*^_−_sin *β* + *μ*^*a*^_+_cos *β*,

where *μ*^*d*^_A_ and *μ*^*d*^_B_ are the diabatic surfaces for 1 ^1^A_2_–X̃ ^1^A_1_ and 1 ^1^B_1_–X̃ ^1^A_1_, respectively, and *μ*^*a*^_±_ are the adiabatic surfaces. These surfaces were likewise taken from Chen *et al.*;^16^ the diabatic *μ*^*d*^_B_ surface is shown in Fig. S1 of their SI.

### 2.2 Nuclear motion calculations

A general variational solution of the time-independent triatomic nuclear motion problem using an exact kinetic energy operator (within the Born–Oppenheimer approximation) was first presented by Sutcliffe and Tennyson.^58–61^ To this end, the nuclear motion program DVR3D^62–64^ was developed to calculate rovibrational energies, wavefunctions and dipole transition moments under this regime for a single electronic state. More recently, the nuclear motion program EVEREST^29^ (electronic, vibrational et rotational energies and spectra for triatomics) was developed as an extension to DVR3D. Primarily, it allows for the treatment of any number of electronic states including couplings (*e.g.* spin–orbit, angular momenta and non-adiabatic) and Renner–Teller effects.

In this study, it was necessary to update EVEREST in order to better treat coupled potential energy surfaces in the diabatic representation. The solution strategy employed by EVEREST (and DVR3D) is to solve the vibrational Schrödinger equation within a time-independent framework for each (*K*,*q*) block of each electronic state, where *K* is the projection of the angular momentum on the body-fixed frame and *q* denotes the AB_2_ permutation symmetry. This is performed within a discrete variable representation (DVR). The rotational motion is subsequently solved for within this vibrational basis set. In the original implementation of EVEREST, all coupling terms were included at the rotational step. Now, off-diagonal potential energy surfaces can be included during the diagonalisation procedure at the vibrational step. This new implementation diagonalises the diabatically-coupled states together, with the off-diagonal coupling surface represented on the DVR grid, for each (*K*,*q*) block. This produces an improved contracted vibrational basis that explicitly includes non-adiabatic mixing to better treat the rotational motion. This is important as the coupling acts strongly across the seam of conical intersections, and thus strongly mixes the vibrational solutions for the bound-like 1 ^1^B_1_ progression and the 1 ^1^A_2_ continuum.

The EVEREST calculations were performed in this work using 30 sinc radial functions for *r*_1,2_ ∈ [1.5,6.0] *a*_0_ and 60 associated Legendre angular basis functions for *θ* ∈ [0,180]° in a symmetrised Radau coordinate system. A vibrational matrix dimension of 20 000 was used with an energy threshold of 80 000 cm^−1^. The rotational matrix was constructed with maximum dimensions of 30 000 and 40 000 for the adiabatic and diabatic representations, respectively. The lowest energy vibrational eigenfunctions allowed within each symmetry block were retained at the rotational step. For application in local thermodynamic equilibrium (LTE) conditions, energy levels were calculated up to *J* = 15 to recover 99.9% of the partition function^44^ at temperature 295 K. Further, at 295 K, 99.995% of the partition function is converged with 519 ground state energy levels below 2500 cm^−1^. Additional information on the computational details and discussion of energy level convergence is given in Section 3.4.

### 2.3 Spectral calculations

All cross sections were computed using ExoCross.^65^ For a transition between initial i and final f discrete states, the line intensity is given by16
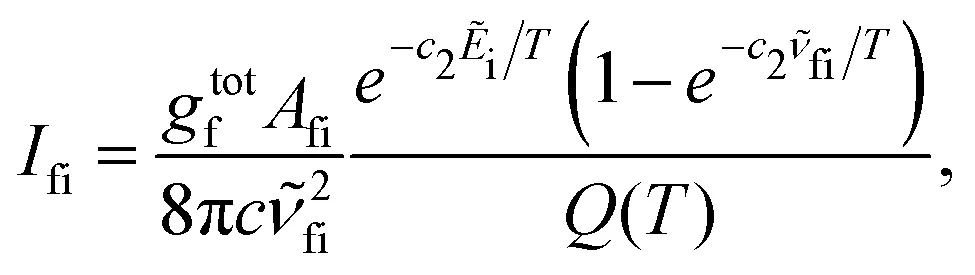
where *g*^tot^ = *g*^ns^(2*J*+ 1) is the total degeneracy factor, *g*^ns^ is the nuclear spin statistical weight factor, *A*_fi_ is the Einstein *A* coefficient, *ν̃*_fi_ is the transition wavenumber, *Ẽ* is energy, *T* is temperature, *c*_2_ = *hc*/*k*_B_, and *Q*(*T*) is the partition function expressed as17
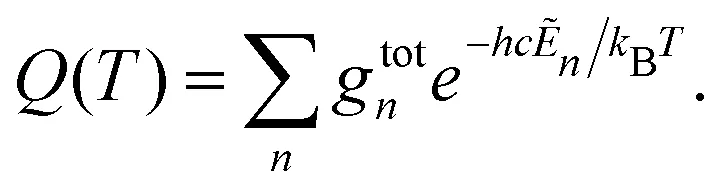


For each spectral line, predissociative lifetime *τ*_pre_ broadening was modelled using a Lorentzian profile given by18
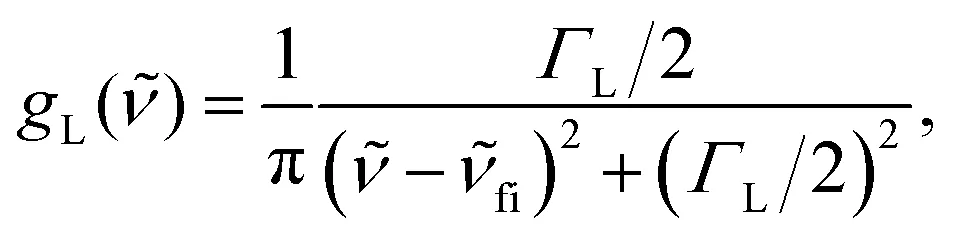
with full-width at half-maximum of19
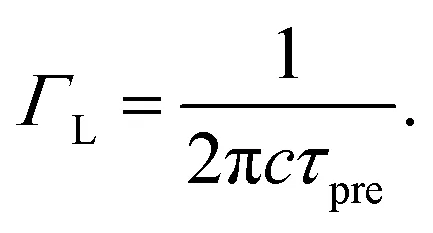


A Gaussian filter was also applied to all smoothed cross sections to model the experimental resolution in this spectral region. For the cross section of Lee *et al.*,^13^ a full-width at half-maximum *Γ*_G_ = 75 cm^−1^ was employed to replicate the resolution of 0.3 nm at 200 nm according to20
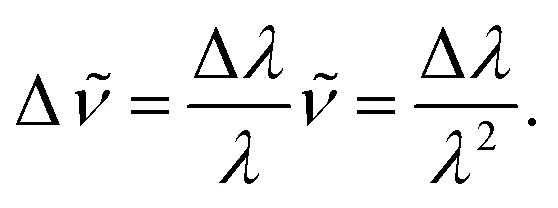


## 3 Results

The photodissociation cross section for H_2_S in its first electronic absorption band was calculated in both the adiabatic and diabatic representations at temperatures 1 and 295 K.

### 3.1 Adiabatic representation

**Fig. 3:**
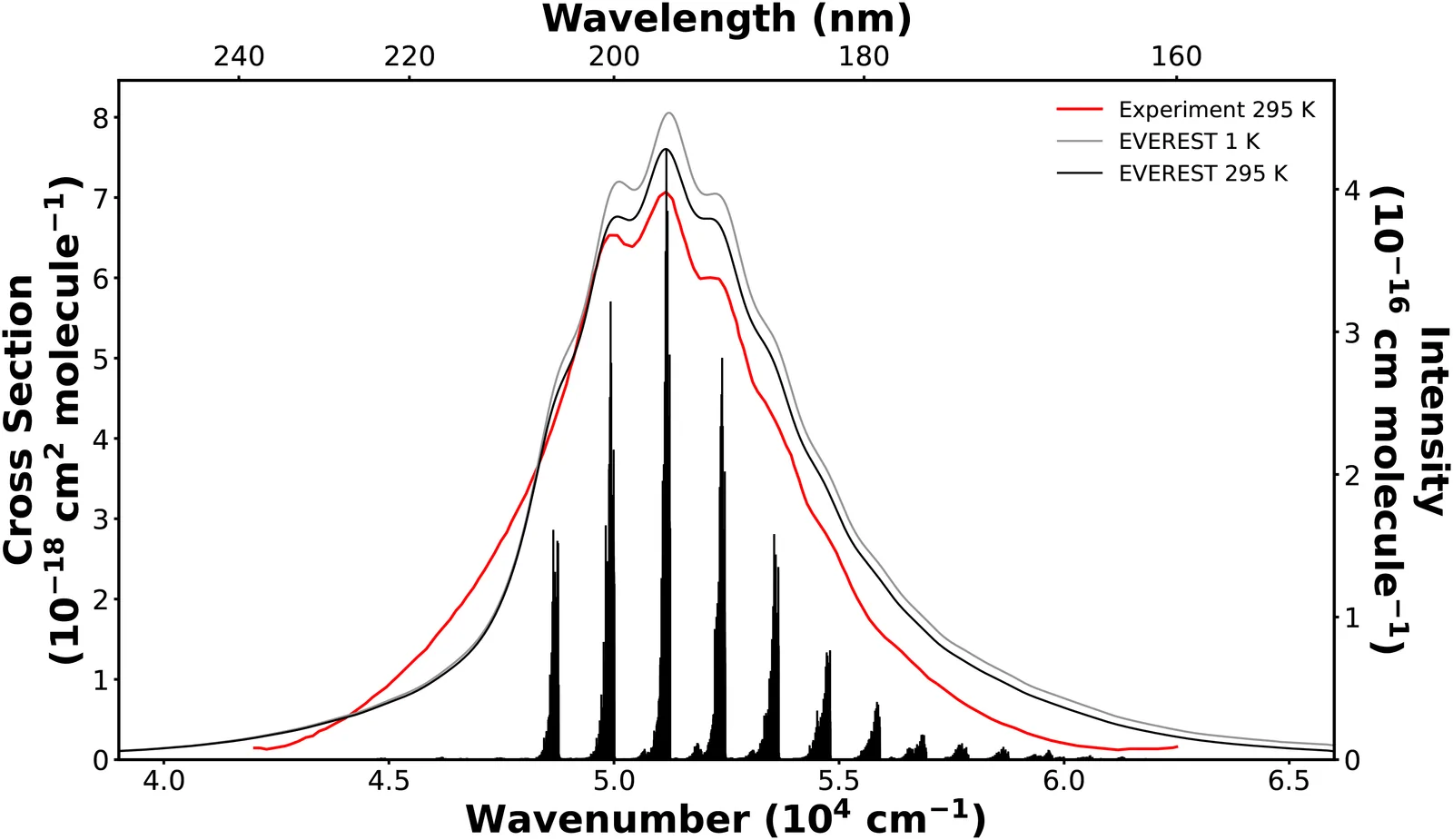
The photodissociation cross sections (left scale) calculated using EVEREST in the adiabatic representation at temperatures 1 and 295 K. The red cross section gives the experimental measurement of Lee *et al.*^13^ at 295 K. The line intensities (right scale) are shown in 1 cm^−1^ bins at 295 K.

The photodissociation cross sections, with line intensities, in the adiabatic representation are shown in Fig. 3 at temperatures 1 and 295 K. Due to the exclusion of any non-adiabatic couplings in this representation, these cross sections effectively give the uncoupled spectrum for the allowed 1 ^1^B_1_–X̃ ^1^A_1_ band system. The upper line positions were shifted by −472 cm^−1^ and Lorentzian profiles of *Γ*_L_ = 1600 cm^−1^ were applied to reproduce the experimental measurements of Lee *et al.*^13^ at 295 K. It can be seen that the diffuse structure is well-produced with an observed progression in the symmetric stretch mode. The low-energy wing below the term energy of the 1 ^1^B_1_ state, however, cannot be fully reproduced with this model. This threshold is associated with the continuum of the 1 ^1^A_2_ state, where no significant intensity is observed below the term energy of the 1 ^1^B_1_ state.

The time-independent approach employed here again gives discrete lines within the continuum that require *ad hoc* broadening. In the adiabatic representation, the 1 ^1^B_1_–X̃ ^1^A_1_ band system is a bound-bound problem that requires lifetime broadening to model predissociation into the 1 ^1^A_2_ continuum. To this end, line broadening was modelled using Lorentzian profiles with full-width at half-maximum *Γ*_L_ = 1600 cm^−1^ to reproduce the diffuse spectral features at 295 K. This value yields a predissociation lifetime *τ*_pre_ = 3.3 fs, which is consistent with the 10^−15^–10^−12^ s estimate of Weiner *et al.*^66^ and <10^−14^ s estimate of van Veen *et al.*^67^ at 193 nm. The order of this predissociative lifetime allows for vibration of the H_2_S molecule before it undergoes dissociation.

### 3.2 Diabatic representation

**Fig. 4:**
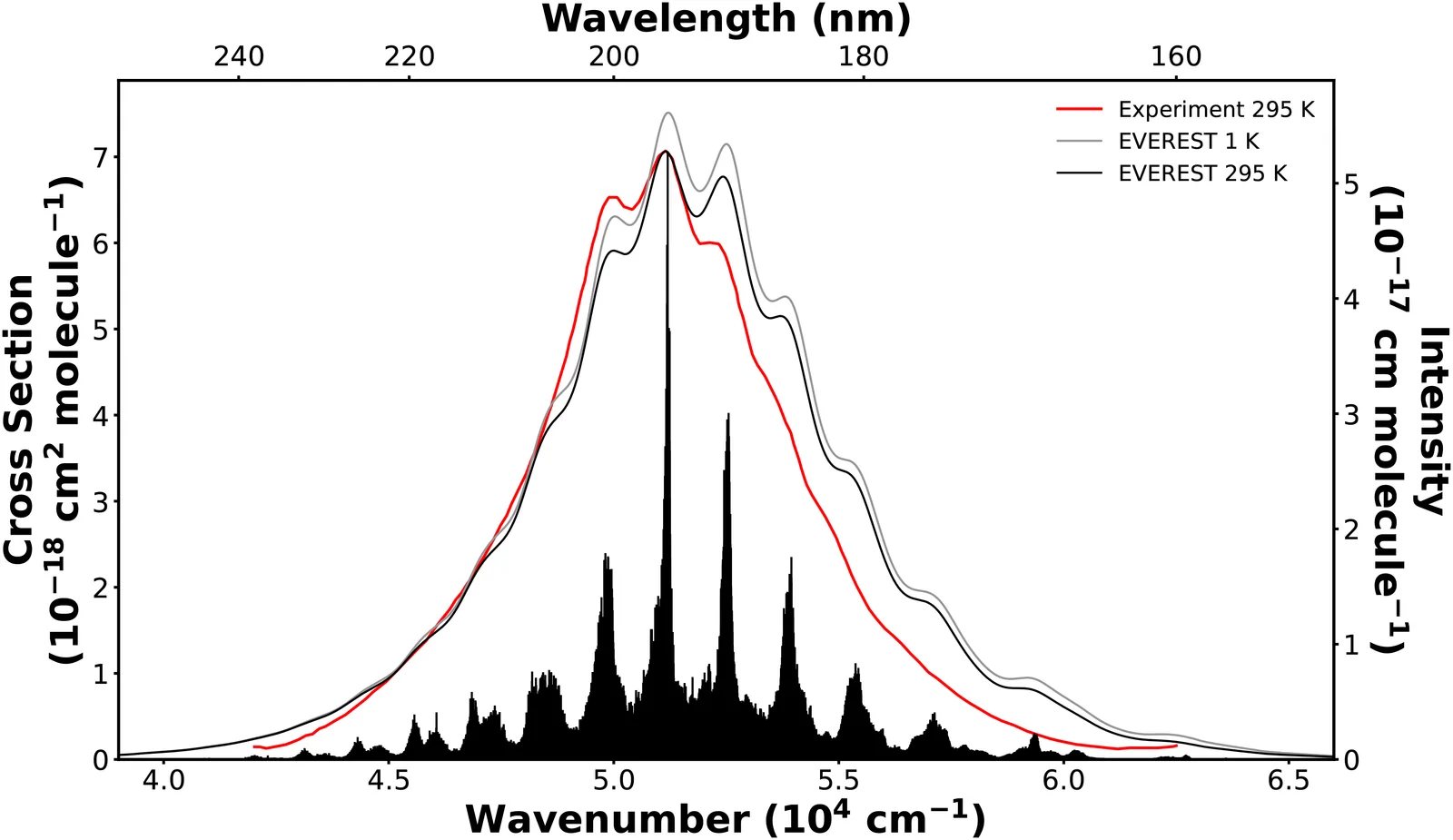
The photodissociation cross sections (left scale) calculated using EVEREST in the diabatic representation at temperatures 1 and 295 K. The red cross section gives the experimental measurement of Lee *et al.*^13^ at 295 K. The line intensities (right scale) are shown in 1 cm^−1^ bins at 295 K.

The photodissociation cross sections, with individual line intensities, in the diabatic representation are shown in Fig. 4 at temperatures 1 and 295 K. The upper line positions were shifted by +279 cm^−1^ and Lorentzian profiles of *Γ*_L_ = 1175 cm^−1^ were applied to reproduce the experimental measurements of Lee *et al.*^13^ at 295 K. It can be seen that the inclusion of non-adiabatic effects through the diabatisation procedure allows for the correct description of the low-energy wing. Using the potential energy and transition dipole moment surfaces of Chen *et al.*,^16^ however, the intensity progression in the diffuse structures is altered.

The failure of the diabatic model to account for the correct diffuse structure from *ab initio* surfaces was noted by Simah *et al.*,^15^ which reflects an insufficient description in the Franck–Condon region. This is particularly sensitive given that the 1 ^1^A_2_ and 1 ^1^B_1_ potential energy surfaces exhibit a conical intersection close to this region. The study of Simah *et al.*^15^ observed a relative increase in the left flank (low-energy region) of their spectra. As such, their initial ground state wavepackets were shortened by 0.05*a*_0_ along the symmetric stretch coordinate to better reproduce the experimental measurements. As detailed by Simah *et al.*,^15^ this moves the wavepacket under a more repulsive region of the excited electronic states. This increases the final population in higher vibrational states associated with the symmetric stretch mode, which increases the relative intensity of the high-energy diffuse peak of the spectrum. Additionally, Simah *et al.*^15^ required a vertical shift of 2.02 nm towards shorter wavelengths in order to reproduce the experimental cross section.

**Fig. 5:**
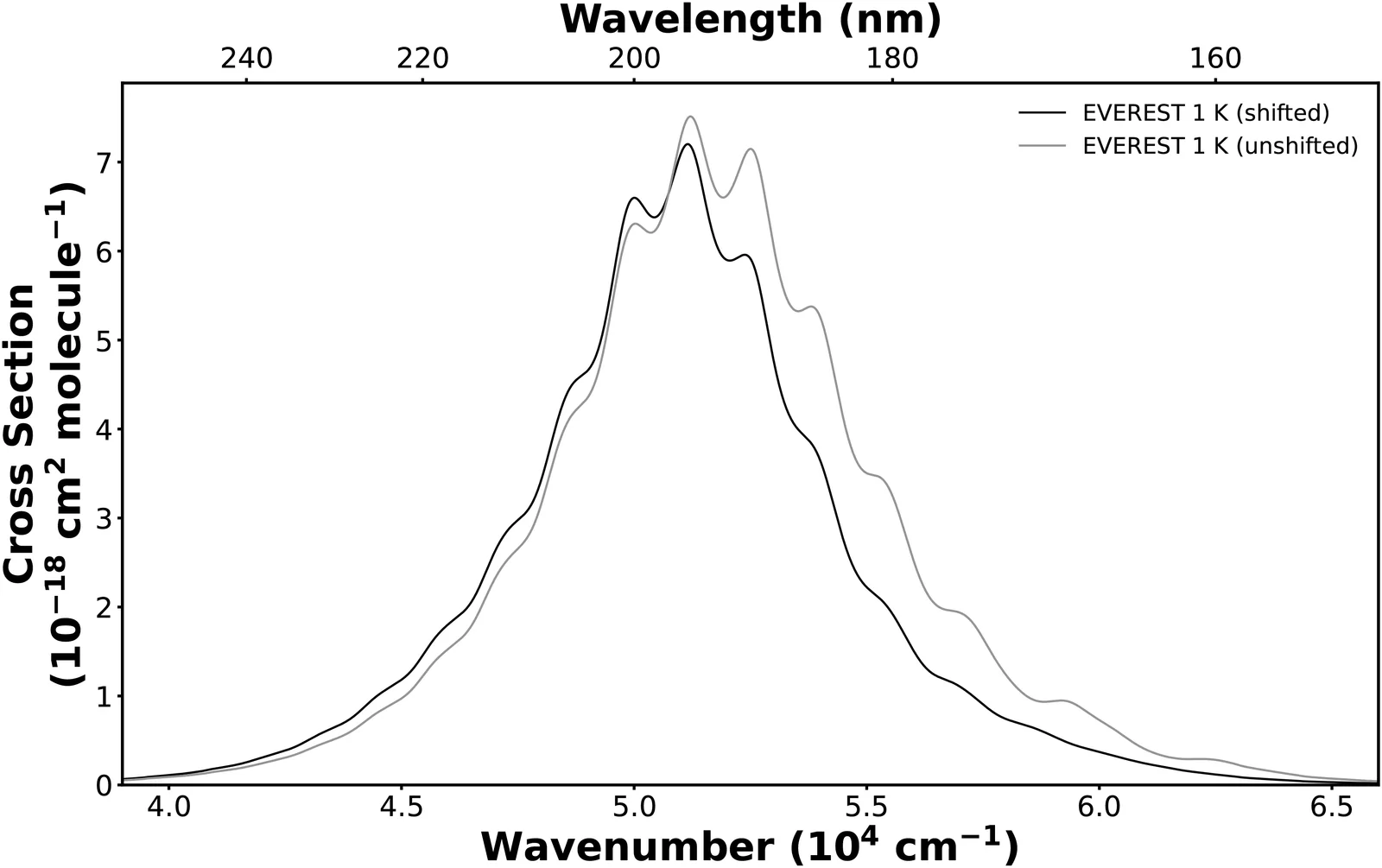
The photodissociation cross sections calculated using EVEREST in the diabatic representation at temperature 1 K. The grey cross section was calculated with the unshifted surfaces of Azzam *et al.*^44^ and Chen *et al.*,^16^ and the black cross section was calculated by shifting the upper potential energy and transition dipole moment surfaces +0.075*a*_0_ along the symmetric stretch coordinate.

In this work, it was found that the right flank (high-energy region) is too intense. In order to test this hypothesis in the diabatic representation, the 1 ^1^A_2_ and 1 ^1^B_1_ potential energy and both transition dipole moment surfaces were shifted along the symmetric stretch coordinate. At temperature 1 K, it was found that a shift of +0.075*a*_0_ better reproduces the intensity progression, as shown in Fig. 5. It should be noted here that the PES-Y0125 potential energy surface of Azzam *et al.*^44^ was used in this work for the ground X̃ ^1^A_1_ electronic state. This was refined to reproduce experimental line positions and is thus known to produce accurate initial wavefunctions within a time-independent variational framework. Consequently, any empirical wavefunction shifts are not expected to match those from the pure *ab initio* studies of Simah *et al.*^15^ and Chen *et al.*,^16^ especially in light of their observed sensitivity to the final cross section in Fig. 5.

The diffuse structure in the final photodissociation cross sections of Chen *et al.*^16^ and Simah *et al.*,^15^ using shifted ground state wavepackets, is consistent with the experimental measurements of Lee *et al.*^13^ within uncertainty. The comparison to these previous *ab initio* time-dependent studies can thus be inferred from the red cross sections in Fig. 3, 4, and 5.

### 3.3 Discussion

It is clear that the diabatic representation yields a better result in the calculation of photodissociation cross sections involving electronic states coupled *via* non-adiabatic effects. It is crucially able to model the low-energy wing while maintaining the diffuse structure of the spectrum using the shifted model. This is important for the calculation of photolysis rates at elevated temperatures for astronomical applications, where the radiation fields of cool stars peak strongly in this near-threshold region.

The adiabatic and diabatic representations do not produce the same result within this framework, which prevents direct comparison of the photodissociation cross sections. The (quasi-)diabatic model treats non-adiabatic effects through the off-diagonal potential energy surface. The adiabatic model, on the other hand, is uncoupled and reflects only the allowed 1 ^1^B_1_–X̃ ^1^A_1_ band system. This is evident from the maximum value of each cross section in Fig. 3 and 4, where there is no intensity redistribution from the central diffuse structure into the low-energy wing *via* state mixing in the adiabatic representation. Nevertheless, the adiabatic model is still useful to probe the vibrational progression along the symmetric stretch coordinate.

Despite the advantages of the diabatic model, it is significantly more computationally expensive. This is reflected in the size of the rotational matrix, even for modest values of *J*, that is required to sufficiently converge the final energy levels. The main limitation is that the 1 ^1^A_2_ and 1 ^1^B_1_ electronic states must be diagonalised together in the diabatic calculations, which yields energy levels that start from the first dissociation limit far from the Franck–Condon region. In order to achieve convergence to a given upper energy, the required matrices are thus approximately double in size as compared to those in the adiabatic representation.

Additionally, both vertical and horizontal shifts of the excited 1 ^1^A_2_ and 1 ^1^B_1_ surfaces indicate that the model of Chen *et al.*^16^ could be further improved for application to the time-independent variational framework presented here. The vertical shift corrects for the error in the vertical excitation energy, whereas the horizontal shift controls the relative intensity progression of the diffuse structures. Additionally, as is evident from Fig. 2, the excited potential energy surfaces do not cross in the diabatic representation such that electronic character is preserved. For an improved time-independent treatment, these concerns should be addressed with new fitted potential energy and dipole moment surfaces. A spectroscopic model that can better reproduce the experimental measurements at temperature 295 K will be the subject of future work, followed by the calculation of temperature-dependent cross sections as required for astronomical studies.

The *ad hoc* line broadening also leads to variations in the simulated cross sections, although there is no difference in integrated intensity for normalised line profiles. A Lorentzian broadening profile was chosen to model the predissociative mechanism responsible for H_2_S dissociation in this band, where the full-width at half-maximum is consistent with lifetime measurements reported in the literature. It should be noted that the photolysis rates calculated from these cross sections are insensitive to the broadening profile, where the redistributed flux experiences a similar photon flux for a smooth radiation field.^28^ Additionally, this treatment assumes that the line broadening is rotationally-independent such that the same parameter can be used at any temperature (*e.g.* at 295 K, in order to reproduce the experimental cross section). This approximation is appropriate due to the large values required compared to the rotational spacing, which smears the discrete structure into one continuum.

### 3.4 Convergence

For a given uncoupled bound potential energy surface, the rovibrational energy levels of a triatomic molecule are capable of being converged to 0.000005 cm^−1^ between different nuclear motion programs.^68^ This typically requires moderate matrix sizes (of order 1000, depending on the molecule and coordinate system used), and becomes highly efficient with an optimised set of Morse-like radial basis functions.

For a repulsive potential energy surface used to describe photodissociative processes, however, the convergence of discrete energy levels sampled within the continuum presents a different challenge. The objective in this case is to converge enough energy levels with sufficient density below a given energy in order to reliably model the continuum. The achievement of spectroscopic accuracy (sub-cm^−1^) is not required in this case due to the large *ad hoc* smoothing parameters employed. As discussed by Pezzella *et al.*,^28,36^ sinc basis functions are preferable for the radial coordinates given that they uniformly sample the bond lengths *r*_1,2_ ∈ [*r*_min_,*r*_max_]. Along the dissociative coordinate, this is particularly important to account for behaviour in continuum regions far from equilibrium. For this reason, sinc basis sets are more difficult to converge than Morse-like basis sets given that they are not localised near the equilibrium geometry of a bound potential well. Additionally, within a time-independent variational framework, the density of states is dependent on the radial box size in analogy to the “particle-in-a-box” model problem. This requires the diagonalisation of successively larger rotational matrices, approximately proportional to *J* + 1, in order to converge levels below a required energy.

Additionally, using this implementation, the treatment of a repulsive potential energy surface is challenging as energy levels are converged from the global minimum, *i.e.* the dissociation limit. These lowest states are typically far from the Franck–Condon region and thus have transitions of negligible intensity in the resulting cross section. For the first electronic absorption band of H_2_S, the minimum energy of the 1 ^1^A_2_ potential energy surface is around 33 890 cm^−1^, whereas the spectrum is centred at 51 150 cm^−1^. In the diabatic representation, this issue is exacerbated as the two excited electronic states must be diagonalised together, and the resonance structure of the 1 ^1^B_1_ state cannot be isolated from the continuum as for the adiabatic model. A filter diagonalisation approach^69^ could be employed to resolve this issue by selectively computing eigenfunctions in the desired spectral region; this will be explored in a future work.

As per above, 30 sinc radial and 60 associated Legendre angular functions were selected for the vibrational basis set in this work. A final rotational matrix size of 30 000 and 40 000 was then used for each (*J*,*p*,*q*) block in the adiabatic and diabatic representations, respectively. These computational parameters were found to correctly model the photodissociation cross section while remaining computationally feasible for investigation of the underlying spectroscopic model, as well as the new diabatic implementation in EVEREST. Upon determination of a final set of potential energy and dipole moment surfaces, these parameters will be optimised to compute temperature-dependent photodissociation cross sections in the diabatic representation for high *J* energy levels. At temperature 2000 K, for example, rovibronic calculations will need to be performed up to *J* = 40 in order to converge the partition function in LTE conditions.

### 3.5 Photolysis rates

The final objective underpinned by this direction of study is the determination of photolysis rates for H_2_S in its first electronic absorption band as a function of molecular temperature. These rates will prove crucial, for example, in the reassessment of atmospheric sulphur chemical cycles in the hot Jupiter exoplanet WASP-39b,^53^ which has been used to explain the presence of SO_2_*via* successive oxidation of H_2_S.

For a given dissociative process, the photolysis rate is given by21
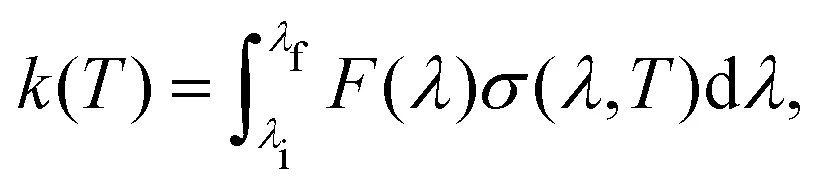
where *λ* is wavelength, *T* is the molecular temperature, *F*(*λ*) is the photon flux, and *σ*(*λ*,*T*) is the temperature-dependent photodissociation cross section.

In Table 1, photolysis rates computed using the adiabatic and shifted diabatic cross sections at molecular temperature *T* = 1 K are presented for several astronomical radiation fields. These were calculated in the 42 500–62 500 cm^−1^ spectral region. Each cross section was scaled to match the maximum experimental value of Lee *et al.*,^13^ and the Leiden normalisation scheme^5^ was employed such that the integrated energy intensity of each field is scaled to match that of the interstellar radiation field of Draine.^70^ These scalings allow for the wavelength-dependent effects of each cross section and radiation field to be assessed. For the constant, interstellar, and hot stellar (*i.e.* 10 000 K and 20 000 K black-body) radiation fields, the photolysis rates are comparable between the adiabatic and diabatic representations. The diabatic photolysis rates are expected to be larger due to the intensity contribution from the low-energy wing. Under the 4000 K black-body radiation field strongly peaked in this low-energy threshold region, however, the diabatic photolysis rate is significantly larger than that calculated in the adiabatic representation.

**Table 1:** The photolysis rates (s^−1^) computed at molecular temperature *T* = 1 K for the adiabatic and shifted diabatic cross sections. These are provided for several radiation fields using the Leiden normalisation convention

Radiation field	Adiabatic	Diabatic
*F*(*λ*) = 1	1.84 × 10^−16^	1.93 × 10^−16^
Interstellar	2.87 × 10^−10^	2.99 × 10^−10^
4000 K BB	3.62 × 10^−09^	4.77 × 10^−09^
10 000 K BB	1.03 × 10^−09^	1.14 × 10^−09^
20 000 K BB	3.98 × 10^−10^	4.14 × 10^−10^

With an increase in molecular temperature, the populations of higher energy states in the initial ensemble increase, and it accordingly requires less energy for a transition to exceed the dissociation limit. It is thus expected that the low-energy wing will increase with temperature, which would substantially increase the photolysis rate under irradiation from cool stellar fluxes. A complete analysis of these effects will again be the subject of future work upon determination of a final spectroscopic model and improvement of convergence properties for high *J* calculations.

## 4 Conclusions

This work presents photodissociation cross sections for H_2_S in its first electronic absorption band at temperatures 1 and 295 K. These were calculated in both the adiabatic and diabatic representations using a time-independent variational framework, where the EVEREST nuclear motion program was updated to facilitate the diabatic calculations.

The coupled diabatic model emphasises that non-adiabatic effects are essential to correctly model photodissociative processes in the vicinity of a conical intersection. This is particularly evident in the threshold region characterised by the low-energy wing of the spectrum. Under cool stellar radiation fields, this threshold region is the most important feature for calculation of the associated photolysis rate, where black-body fluxes peak in intensity.

The future direction of this work is towards a complete set of temperature-dependent photodissociation cross sections for H_2_S in its first electronic absorption band. The failure of the diabatic model to correctly account for the intensity progression in the diffuse structures indicates that the existing set of potential energy and transition dipole moment surfaces could be improved for time-independent variational calculations. This study has shown that simple vertical and horizontal shifts of the excited *ab initio* spectroscopic model can better reproduce experimental measurements. It will thus be favourable to better explore such shifts, as well as morphing and experimental refinement, of the upper surfaces to arrive at a final set of photodissociation cross sections at elevated temperatures.

The diabatic methodology established in this work, now possible using EVEREST, hence serves as a framework to calculate photodissociation cross sections for triatomic molecules with coupled electronic states. A coupled three-state model is currently being developed for the first two electronic absorption bands of H_2_O below 80 000 cm^−1^. This will also allow for an investigation of near-ultraviolet photoabsorption processes with the inclusion of non-adiabatic couplings and Renner–Teller effects. Other molecules of astronomical interest include SO_2_ and CO_2_.

## Conflicts of interest

There are no conflicts to declare.

## Supplementary Material

CP-028-D6CP02139E-s001

## Data Availability

All data are contained within the article. Supplementary information (SI) is available. Supplementary information: adiabatic and diabatic cross sections calculated at temperature *T* = 295 K in the 42 500–62 500 cm^−1^ spectral region. See DOI: https://doi.org/10.1039/d6cp02139e.

## References

[cit1] van Dishoeck E. F. and Visser R., in Molecular Photodissociation, John Wiley & Sons, Ltd, 2014, ch. 4, pp. 229–254

[cit2] Turco R. P. (1975). Geophys. Surv..

[cit3] Wayne R. P. (1993). J. Geophys. Res.: Planets.

[cit4] Eddington A. S. (1928). Mon. Not. R. Astron. Soc..

[cit5] Heays A. N., Bosman A. D., van Dishoeck E. F. (2017). Astron. Astrophys..

[cit6] Hrodmarsson H. R., van Dishoeck E. F. (2023). Astron. Astrophys..

[cit7] van Harrevelt R., van Hemert M. C. (2000). J. Chem. Phys..

[cit8] van Harrevelt R., van Hemert M. C. (2000). J. Chem. Phys..

[cit9] Harrevelt R. V., Hemert M. C. V. (2001). J. Chem. Phys..

[cit10] Yuan K., Dixon R. N., Yang X. (2011). Acc. Chem. Res..

[cit11] Hu X., Zhou L., Xie D. (2018). Wiley Interdiscip. Rev.: Comput. Mol. Sci..

[cit12] Mota R., Parafita R., Giuliani A., Hubin-Franskin M.-J., Lourenço J., Garcia G., Hoffmann S., Mason N., Ribeiro P., Raposo M., Limão-Vieira P. (2005). Chem. Phys. Lett..

[cit13] Lee L. C., Wang X., Suto M. (1987). J. Chem. Phys..

[cit14] Heumann B., Weide K., Düren R., Schinke R. (1993). J. Chem. Phys..

[cit15] Simah D., Hartke B., Werner H.-J. (1999). J. Chem. Phys..

[cit16] Chen J., Zhang H., Zhou L., Hu X., Xie D. (2023). Phys. Chem. Chem. Phys..

[cit17] Dixon R. N., Marston C. C., Balint-Kurti G. G. (1990). J. Chem. Phys..

[cit18] Guest M., Rodwell W. (1976). Mol. Phys..

[cit19] Roberge R., Salahub D. R. (1979). J. Chem. Phys..

[cit20] Kulander K. C. (1984). Chem. Phys. Lett..

[cit21] Rauk A., Collins S. (1984). J. Mol. Spectrosc..

[cit22] Diercksen G., Langhoff P. (1987). J. Chem. Phys..

[cit23] Halonen L., Carrington T. (1988). J. Chem. Phys..

[cit24] Weide K., Staemmler V., Schinke R. (1990). J. Chem. Phys..

[cit25] Heumann B., Düren R., Schinke R. (1991). Chem. Phys. Lett..

[cit26] Heller E. J. (1978). J. Chem. Phys..

[cit27] Pezzella M., Mitev G., Yurchenko S. N., Tennyson J., Mitrushchenkov A. O. (2024). Phys. Chem. Chem. Phys..

[cit28] Pezzella M., Yurchenko S. N., Tennyson J. (2021). Phys. Chem. Chem. Phys..

[cit29] Mitrushchenkov A. O. (2012). J. Chem. Phys..

[cit30] Tennyson J., Yurchenko S. N. (2012). Mon. Not. R. Astron. Soc..

[cit31] Tennyson J., Yurchenko S. N., Al-Refaie A. F., Barton E. J., Chubb K. L., Coles P. A., Diamantopoulou S., Gorman M. N., Hill C., Lam A. Z., Lodi L., McKemmish L. K., Na Y., Owens A., Polyansky O. L., Rivlin T., Sousa-Silva C., Underwood D. S., Yachmenev A., Zak E. (2016). J. Mol. Spectrosc..

[cit32] Tennyson J., Yurchenko S. N., Al-Refaie A. F., Clark V. H. J., Chubb K. L., Conway E. K., Dewan A., Gorman M. N., Hill C., Lynas-Gray A. E., Mellor T., McKemmish L. K., Owens A., Polyansky O. L., Semenov M., Somogyi W., Tinetti G., Upadhyay A., Waldmann I., Wang Y., Wright S., Yurchenko O. P. (2020). J. Quant. Spectrosc. Radiat. Transfer.

[cit33] Tennyson J., Yurchenko S. N., Zhang J., Bowesman C. A., Brady R. P., Buldyreva J., Chubb K. L., Gamache R. R., Gorman M. N., Guest E. R., Hill C., Kefala K., Lynas-Gray A. E., Mellor T. M., McKemmish L. K., Mitev G. B., Mizus I. I., Owens A., Peng Z., Perri A. N., Pezzella M., Polyansky O. L., Qu Q., Semenov M., Smola O., Sokolov A., Somogyi W., Upadhyay A., Wright S. O. M., Zobov N. F. (2024). J. Quant. Spectrosc. Radiat. Transfer.

[cit34] Yurchenko S. N., Lodi L., Tennyson J., Stolyarov A. V. (2016). Comput. Phys. Commun..

[cit35] Ni Q.-H., Hill C., Yurchenko S. N., Pezzella M., Fateev A. Z., Qin Z., Venot O., Tennyson J. (2025). RAS Tech. Instrum..

[cit36] Pezzella M., Yurchenko S. N., Tennyson J. (2022). Mon. Not. R. Astron. Soc..

[cit37] Mitev G. B., Yurchenko S. N., Tennyson J. (2024). J. Chem. Phys..

[cit38] Mitev G. B., Pezzella M., Bowesman C. A., Zhang J., Yurchenko S. N., Tennyson J. (2025). Mon. Not. R. Astron. Soc..

[cit39] Uhlíková T., Yurchenko S. N., Perri A. N., Tennyson J., Kim G.-S. (2025). J. Chem. Phys..

[cit40] Perri A. N., Uhlíková T., Kim G.-S., Yurchenko S. N., Tennyson J. (2026). Mon. Not. R. Astron. Soc..

[cit41] Sokolov A., Brady R. P., Yurchenko S. N., Tennyson J. (2025). Mon. Not. R. Astron. Soc..

[cit42] Sokolov A., Yurchenko S. N., Tennyson J. (2026). Phys. Chem. Chem. Phys..

[cit43] Brady R. P., Sokolov A., Yurchenko C. A. B. S. N., Tennyson J. (2026). Mon. Not. R. Astron. Soc..

[cit44] Azzam A. A. A., Yurchenko S. N., Tennyson J., Naumenko O. V. (2016). Mon. Not. R. Astron. Soc..

[cit45] Werner H., Knowles P. J., Knizia G., Manby F. R., Schütz M. (2012). Wiley Interdiscip. Rev.: Comput. Mol. Sci..

[cit46] Werner H.-J., Knowles P. J., Manby F. R., Black J. A., Doll K., HeÃŸelmann A., Kats D., Köhn A., Korona T., Kreplin D. A., Ma Q., Miller I., Thomas F., Mitrushchenkov A., Peterson K. A., Polyak I., Rauhut G., Sibaev M. (2020). J. Chem. Phys..

[cit47] Knowles P. J., Werner H.-J. (1985). Chem. Phys. Lett..

[cit48] Werner H.-J., Knowles P. J. (1985). J. Chem. Phys..

[cit49] Shiozaki T., Werner H.-J. (2011). J. Chem. Phys..

[cit50] Shiozaki T., Knizia G., Werner H.-J. (2011). J. Chem. Phys..

[cit51] Peterson K. A., Adler T. B., Werner H.-J. (2008). J. Chem. Phys..

[cit52] Alderson L., Wakeford H. R., Alam M. K., Batalha N. E., Lothringer J. D., Adams Redai J., Barat S., Brande J., Damiano M., Daylan T., Espinoza N., Flagg L., Goyal J. M., Grant D., Hu R., Inglis J., Lee E. K. H., Mikal-Evans T., Ramos-Rosado L., Roy P.-A., Wallack N. L., Batalha N. M., Bean J. L., Benneke B., Berta-Thompson Z. K., Carter A. L., Changeat Q., Colón K. D., Crossfield I. J. M., Désert J.-M., Foreman-Mackey D., Gibson N. P., Kreidberg L., Line M. R., López-Morales M., Molaverdikhani K., Moran S. E., Morello G., Moses J. I., Mukherjee S., Schlawin E., Sing D. K., Stevenson K. B., Taylor J., Aggarwal K., Ahrer E.-M., Allen N. H., Barstow J. K., Bell T. J., Blecic J., Casewell S. L., Chubb K. L., Crouzet N., Cubillos P. E., Decin L., Feinstein A. D., Fortney J. J., Harrington J., Heng K., Iro N., Kempton E. M.-R., Kirk J., Knutson H. A., Krick J., Leconte J., Lendl M., MacDonald R. J., Mancini L., Mansfield M., May E. M., Mayne N. J., Miguel Y., Nikolov N. K., Ohno K., Palle E., Parmentier V., Petit dit de la Roche D. J. M., Piaulet C., Powell D., Rackham B. V., Redfield S., Rogers L. K., Rustamkulov Z., Tan X., Tremblin P., Tsai S.-M., Turner J. D., de Val-Borro M., Venot O., Welbanks L., Wheatley P. J., Zhang X. (2023). Nature.

[cit53] Tsai S.-M., Lee E. K. H., Powell D., Gao P., Zhang X., Moses J., Hébrard E., Venot O., Parmentier V., Jordan S., Hu R., Alam M. K., Alderson L., Batalha N. M., Bean J. L., Benneke B., Bierson C. J., Brady R. P., Carone L., Carter A. L., Chubb K. L., Inglis J., Leconte J., Line M., López-Morales M., Miguel Y., Molaverdikhani K., Rustamkulov Z., Sing D. K., Stevenson K. B., Wakeford H. R., Yang J., Aggarwal K., Baeyens R., Barat S., de Val-Borro M., Daylan T., Fortney J. J., France K., Goyal J. M., Grant D., Kirk J., Kreidberg L., Louca A., Moran S. E., Mukherjee S., Nasedkin E., Ohno K., Rackham B. V., Redfield S., Taylor J., Tremblin P., Visscher C., Wallack N. L., Welbanks L., Youngblood A., Ahrer E.-M., Batalha N. E., Behr P., Berta-Thompson Z. K., Blecic J., Casewell S. L., Crossfield I. J. M., Crouzet N., Cubillos P. E., Decin L., Désert J.-M., Feinstein A. D., Gibson N. P., Harrington J., Heng K., Henning T., Kempton E. M.-R., Krick J., Lagage P.-O., Lendl M., Lothringer J. D., Mansfield M., Mayne N. J., Mikal-Evans T., Palle E., Schlawin E., Shorttle O., Wheatley P. J., Yurchenko S. N. (2023). Nature.

[cit54] Powell D., Feinstein A. D., Lee E. K. H., Zhang M., Tsai S.-M., Taylor J., Kirk J., Bell T., Barstow J. K., Gao P., Bean J. L., Blecic J., Chubb K. L., Crossfield I. J. M., Jordan S., Kitzmann D., Moran S. E., Morello G., Moses J. I., Welbanks L., Yang J., Zhang X., Ahrer E.-M., Bello-Arufe A., Brande J., Casewell S. L., Crouzet N., Cubillos P. E., Demory B.-O., Dyrek A., Flagg L., Hu R., Inglis J., Jones K. D., Kreidberg L., López-Morales M., Lagage P.-O., Meier Valdés E. A., Miguel Y., Parmentier V., Piette A. A. A., Rackham B. V., Radica M., Redfield S., Stevenson K. B., Wakeford H. R., Aggarwal K., Alam M. K., Batalha N. M., Batalha N. E., Benneke B., Berta-Thompson Z. K., Brady R. P., Caceres C., Carter A. L., Désert J.-M., Harrington J., Iro N., Line M. R., Lothringer J. D., MacDonald R. J., Mancini L., Molaverdikhani K., Mukherjee S., Nixon M. C., Oza A. V., Palle E., Rustamkulov Z., Sing D. K., Steinrueck M. E., Venot O., Wheatley P. J., Yurchenko S. N. (2024). Nature.

[cit55] Brady R. P., Drury C., Yurchenko S. N., Tennyson J. (2024). J. Chem. Theory Comput..

[cit56] Baer M. (2002). Phys. Rep..

[cit57] Wolfgang Domcke H. K. and Yarkony D. R., Conical intersections: electronic structure, dynamics & spectroscopy, World Scientific Publishing Company, 2004

[cit58] Tennyson J., Sutcliffe B. T. (1982). J. Chem. Phys..

[cit59] Sutcliffe B. T., Tennyson J. (1986). Mol. Phys..

[cit60] Sutcliffe B. T., Tennyson J. (1987). J. Chem. Soc..

[cit61] Sutcliffe B. T., Tennyson J. (1991). Int. J. Quantum Chem..

[cit62] Henderson J. R., Le Sueur C. R., Tennyson J. (1993). Comput. Phys. Commun..

[cit63] Tennyson J., Henderson J. R., Fulton N. G. (1995). Comput. Phys. Commun..

[cit64] Tennyson J., Kostin M. A., Barletta P., Harris G. J., Polyansky O. L., Ramanlal J., Zobov N. F. (2004). Comput. Phys. Commun..

[cit65] Yurchenko S. N., Al-Refaie A. F., Tennyson J. (2018). Astron. Astrophys..

[cit66] Weiner B. R., Levene H. B., Valentini J. J., Baronavski A. P. (1989). J. Chem. Phys..

[cit67] van Veen G., Mohamed K., Baller T., de Vries A. (1983). Chem. Phys..

[cit68] Ovsyannikov R. I., Mizus I. I., Perri A. N., Tennyson J., Yurchenko S. N., Mitrushchenkov A., Zobov N. F., Rogov M. A., Polyansky O. L. (2026). J. Mol. Spectrosc..

[cit69] Mandelshtam V. A., Grozdanov T. P., Taylor H. S. (1995). J. Chem. Phys..

[cit70] Draine B. T. (1978). Astrophys. J., Suppl. Ser..

